# A 3D patternoid model for the reproducible characterization of invasive phenotypes and drug sensitivity in PDAC†

**DOI:** 10.1039/d5lc00203f

**Published:** 2025-05-26

**Authors:** Sophie C. Kurzbach, Violetta Carvajal-Heckele, Tetsuhiko F. Teshima, Maximilian Reichert, Andreas R. Bausch

**Affiliations:** a TUM School of Natural Sciences, Department of Bioscience, Heinz Nixdorf Chair in Biophysical Engineering of Living Matter, Technical University of Munich 85748 Garching Germany abausch@tum.de; b Center for Protein Assemblies (CPA), Technical University of Munich 85747 Garching Germany; c Medical and Health Informatics Laboratories, NTT Research Incorporated Sunnyvale CA 94085 USA; d Munich Institute of Biomedical Engineering, Technische Universitat Munchen 85748 Garching Germany; e Translational Pancreatic Cancer Research Center, TUM School of Medicine and Health, Department of Clinical Medicine Research Center, TUM School of Medicine and Health, University Hospital, Technical University of Munich Germany; f Center for Organoid Systems (COS), Technical University Munich (TUM) 85747 Garching Germany; g German Cancer Consortium (DKTK), partner site Munich, a partnership between DKFZ and University Hospital Klinikum rechts der Isar Germany; h Bavarian Cancer Research Center (BZKF) Munich Germany

## Abstract

Pancreatic ductal adenocarcinoma (PDAC) is a highly invasive and heterogeneous malignancy, posing challenges for reproducible modeling and functional phenotypic analysis. To address these limitations, we developed a standardized 3D patternoid platform using collagen-based microcavity arrays to enhance organoid formation consistency and quantify subtype-specific invasion mechanisms. We utilized murine primary PDAC cells stratified by epithelial–mesenchymal transition (EMT) into three subtypes: epithelial (*E-9591*), hybrid EMT (*Mlow-8028*), and mesenchymal (*M-16992*). The platform's sensitivity was verified by a strong correlation between EMT scores and invasive phenotypes, as well as responses to physiological concentrations of the protease inhibitor batimastat. Key invasion parameters—including invasive area, maximum invasion distance, and branching complexity—were measured under both genomic and drug-induced conditions. The platform demonstrated high inter-organoid reproducibility, with precise control over initial cell numbers ensuring batch-to-batch comparability. Invasion dynamics analysis revealed that epithelial cells (*E-9591*) primarily relied on spatial constraints within the microcavity to invade. Batimastat drug sensitivity assays further distinguished invasion dependencies of the mesenchymal subtypes, confirming that *M-16992* patternoids exhibit a stronger sensitivity towards MMP inhibition compared to *Mlow-8028* patternoids. Concurrentlty, both subtypes experienced a shift towards epithelial-like spatial constraint triggered invasion morphology, reflecting the plasticity of PDAC invasiveness. This scalable and adaptable 3D patternoid platform enables high-throughput analysis of invasive behaviors and therapeutic responses, offering significant potential for preclinical cancer research and personalized medicine.

## Abstract 2

A reproducible 3D patternoid platform enabling high-throughput analysis of PDAC invasion dynamics and drug sensitivities with biomimetic microcavities.

## Graphical abstract

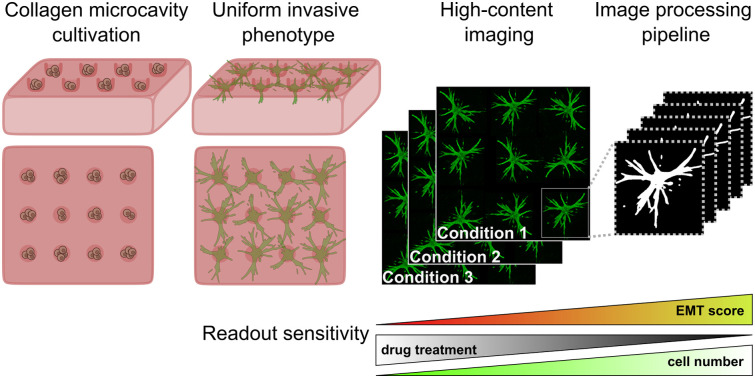

## 1 Introduction

Pancreatic ductal adenocarcinoma (PDAC) is among the most aggressive malignancies, with late-stage diagnosis and pronounced inter- and intratumoral heterogeneity contributing to its poor prognosis.^1–3^ Existing clinical approaches for therapeutic selection primarily focus on genetic profiling and histopathology; however, these methods fail to capture functional aspects of tumor behavior, such as invasive phenotypes and therapeutic resistance.^4^ To address this limitation, three-dimensional (3D) patient-derived organoid (PDO) systems have emerged as promising tools for modeling tumor dynamics and screening drug sensitivity in the context of individual patient profiles. These systems have demonstrated significant potential, particularly in generating chemograms that correlate drug responses with patient outcomes, highlighting their relevance in precision medicine for multiple cancer types.^5–7^

Most PDO systems currently rely on spheroid-based models cultured in Matrigel domes. These models often face challenges related to reproducibility, such as variability in starting cell numbers and limited control over the microenvironment. As a result, they predominantly yield spherical morphologies, which restrict many analyses to viability assays and can reduce the capacity to explore mechanisms of invasion and metastasis.^8^ To expand the investigative potential of organoid models, Papagyriou *et al.* has established a branched organoid system for murine and human pancreatic cancer. This approach aims to more closely mimic key features of the pancreatic cancer phenotype and has been used to study processes such as epithelial-to-mesenchymal transition (EMT) and its role in invasive behavior, both of which are relevant for understanding metastasis and therapy resistance. While this system contributes valuable insights into PDAC heterogeneity, certain aspects of its application—including reproducibility in the context of drug response assessment—remain areas of ongoing refinement.^9,10^

To overcome these limitations, we introduce a 3D patternoid platform that employs biomimetic microcavities to standardize organoid formation. The patternoid model achieves high sensitivity in correlating EMT scores with invasive phenotypes while ensuring low inter-organoid heterogeneity and batch-to-batch reproducibility through precise control of starting cell numbers. Additionally, the defined spatial arrangement of patternoids within the extracellular matrix (ECM) facilitates high-content phenotypic analyses. This platform thus provides robust and reproducible readouts, enabling direct assessment of distinct invasion mechanisms and drug responses, highlighting its potential for precision medicine and preclinical research.

## 2 Results

### 2.1 Development and characterization of PDAC patternoids for invasive phenotype analysis

**Fig. 1:**
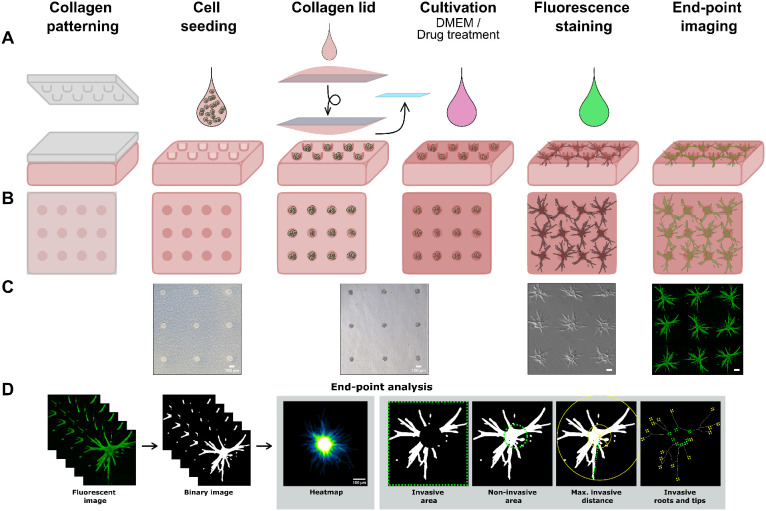
Pipeline for the generation of PDAC patternoids and invasive phenotype characterization. A The collagen is patterned using a PDMS stamp that generates 25 × 26 microcavities in the collagen gel (3 × 4 shown in top view illustration (B) and top view images (C) for simplification), followed by seeding singularized cells into the microcavities. After washing, a collagen lid is added with a glass coverslip, which is subsequently removed before adding the cultivation medium. After 24 h, either a medium change is done or batimastat treatment is performed for 48 h. At the endpoint, patternoids are fluorescence-stained and imaged. D Segmented patternoids (binary image) are used to extract key invasive parameters. Qualitative comparison under varying conditions is performed based on SUM z-projections of binary images of segmented patternoids (heatmaps).

By seeding cells into a defined array of 650 collagen microcavities with a precisely controlled cylindrical shape, we establish a standardized geometric baseline for patternoid development (Fig. 1A–C). This setup minimizes inter-organoid morphological heterogeneity. The surrounding high-concentration collagen matrix was specifically selected to mimic the elevated stiffness of the PDAC tumor microenvironment, a key factor in inducing mechanical cues that drive cancer progression and invasion.^11^

**Fig. 2:**
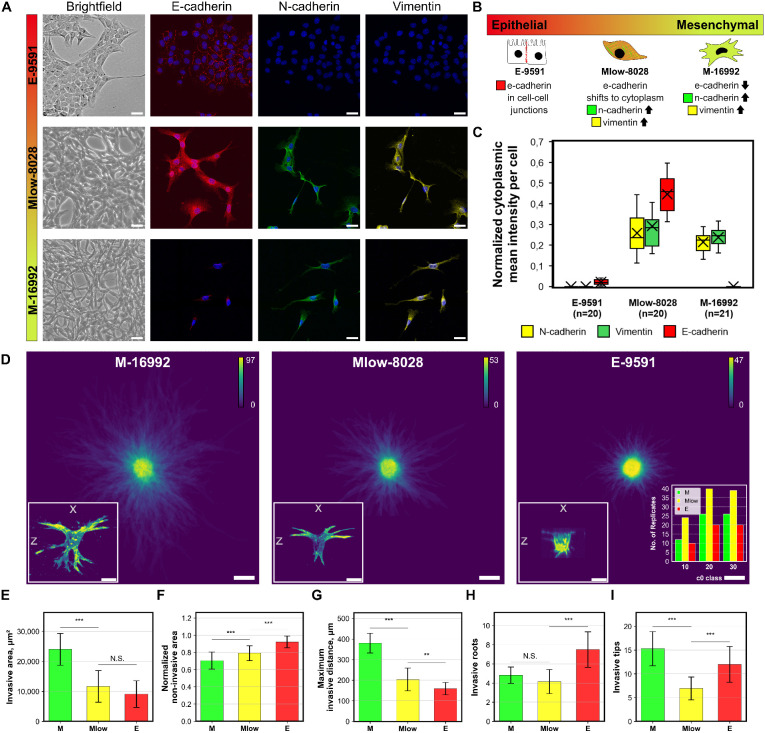
EMT marker expression in PDAC subtypes and reproducible invasive patternoid phenotype. A Representative brightfield (BF) images of PDAC subtypes in adherent cell culture at confluencies around 70–90% show distinct phenotypes. Fluorescence (FLUO) images show localization and expression levels for E- and N-cadherin and vimentin and DAPI (blue) for nucleus localization. To enable a direct comparison across subtypes, brightness settings were kept consistent; this standardization may obscure low-expression signals. These weaker signals are visualized separately in Fig. S7.† Scale bar = 50 μm. BF and FLUO images originate from different experiments. B Illustration of EMT score characteristics in regards of phenotype, EMT marker expression levels and categorization into epithelial/mesenchymal. C Cytoplasmic E-cadherin levels are quantified together with N-cadherin and vimentin for comparability, while specific E-cadherin localization at cell–cell junctions (visible in images) highlights phenotypic differences. D Binary image heatmaps of all patternoid replicates that were used for analysis in E. The number of replicates is indicated in the calibration scale. A representative patternoid 3D reconstruction is presented in the lower left of each heatmap. The *c*_0_ class distribution of the replicates used for each heatmap is shown in the lower right. E–I Quantitative invasive parameters of each PDAC subtype. *N* = 2 independent experiments. The non-invasive area was normalized to the projected area of the circular microcavity (*d* = 100 μm, *A* = 7854 μm^2^). Scale bar = 100 μm.

This approach enables the generation of hundreds of invasive PDAC patternoids within only three days, producing distinct invasive phenotypes (Fig. 2D) based on the genomic profiles of the tumor-derived cells used (Fig. 2A–C). To assess the platform's sensitivity to genomic differences, we utilized primary cancer cells derived from KC mice that developed varying Kras dosages during tumor progression.^12^ These cells were analyzed to characterize subtype-specific invasive phenotypes.

Patternoid characterization was performed using z-projected segmentation analysis to quantify five key invasive parameters (Fig. 1D). The invasive area provides a general measure of invasion extent, while the maximum invasive distance reflects the subtype's invasive potential. Additionally, branching complexity was analyzed by quantifying the number of invasive roots and tips, with the ratio of tips to roots defining the branching factor. The invasive roots number was found to be an indicator for the reliance of PDAC subtypes on spatial constraints as a trigger for invasion, which will be discussed in detail in the subsection 2.5. The non-invasive area represents the area within the microcavity, that is covered by non-invasive cells at day 3.

A crucial criterion for batch comparability and reliable differentiation of PDAC subtypes—based solely on genomically driven phenotypic differences—is the control of starting cell numbers within each microcavity. Despite using the same initial cell density during seeding, variability in the starting cell number may arise due to shear forces during washing steps, leading to uneven cell distribution. While automated handling can reduce variability, inherent statistical fluctuations (*e.g.*, Poisson distribution effects) still affect the initial cell count per microcavity. Consequently, documenting the starting cell number is essential for ensuring reproducibility.

Patternoids were therefore categorized into *c*_0_ classes, defined as tolerance ranges (*c*_0_ ± 10%) around a target starting cell number *c*_0_. This range was experimentally determined to balance sensitivity and practicality in analysis. For the comparison between batches, only patternoid replicates derived from the same *c*_0_ class were used for analysis. Alternatively, patternoid replicates from multiple *c*_0_ classes can be pooled for analysis, as applied for the analysis shown in Fig. 2D and E. When using this approach, maintaining a consistent distribution of *c*_0_ classes across batches is critical to ensure reliable comparisons (Fig. 2D, bottom right).

To precisely control starting cell numbers, we established a documentation step at time point 0. An overview image of each sample was captured within 2 h after seeding (Fig. S6A†), enabling accurate determination of the starting cell number for each patternoid ID. Patternoids meeting strict inclusion criteria were selected for further analysis (see Methods 4 and Fig. S6C–F†). While only 10–25% of seeded microcavities yielded suitable patternoids, this approach significantly reduces variability caused by seeding inconsistencies. As a result, phenotypic readouts accurately reflect genomic differences and drug effects, ensuring high experimental reliability.

Besides the acceptable margin of variability for the definition of the *c*_0_ class, also the definition of the minimum *c*_0_ class and thus the cell seeding density strongly correlates with the heterogeneity of the cell population used. The minimum *c*_0_ class in this study was set at 10; however, for cell populations with increased heterogeneity, such as patient-derived samples, a higher *c*_0_ class may be necessary to ensure reproducibility.

### 2.2 Correlation between EMT score and patternoid invasive phenotypes

To systematically correlate EMT-driven invasion with phenotypic outputs as demonstration for the patternoids platform's utility. Primary cancer cells derived from KC mice—a model representing the cellular heterogeneity observed in PDAC patients—were utilized for this study. These cells, classified into epithelial (*E-9591*), hybrid EMT (*Mlow-8028*), and mesenchymal (*M-16992*) subtypes using bulk RNA-seq,^10^ were analyzed for key EMT markers, including cytoplasmic E-cadherin (E-cadh), N-cadherin (N-cadh), and vimentin, through immunofluorescence staining and confocal imaging (Fig. 2A and 7). A central aim was to determine whether the *Mlow-8028* subtype represents a true hybrid EMT state, characterized by intermediate expression levels of epithelial and mesenchymal markers, or merely a mixture of epithelial and mesenchymal cells.

Using these cell lines, PDAC subtype patternoids were generated to quantify differences in invasive behaviour driven by distinct EMT states. Patternoid replicates for analysis were selected based on the starting cell number *c*_0_ class of 10, 20, and 30. Comparable proportions of each *c*_0_ class were pooled across all PDAC subtypes to ensure consistency and comparability (Fig. 2D, right bottom).

In ***E-9591***, the specific localization of E-cadh in the cell–cell junctions was observed qualitatively (Fig. 2A). No cytoplasmic E-cadh nor mesenchymal marker expression levels further reflect the epithelial character of this subtype (Fig. 2C). The low N-cadh and vimentin levels suggest a rigid cytoskeleton and low adaptability, conferring a minimal EMT score (Fig. 2B) that aligns with the low invasive potential that is observed in the patternoid morphology (Fig. 2D–I).^13^

***Mlow-8028*** is characterized by cytoplasmic E-cadherin and elevated N-cadherin and vimentin levels (Fig. 2A and C), which rises suggestions to a classification as hybrid subtype (Fig. 2B). The cytoplasmic localization of E-cadherin indicates reduced cell–cell adhesion and a partial loss of epithelial integrity, typical of cells that are transitioning along the EMT spectrum. Concurrently, high N-cadherin and vimentin levels introduce mesenchymal properties that enhance cellular flexibility, adaptability, and motility within the ECM (Fig. 2B).^14^ The cellular plasticity enables localized invasion while retaining some epithelial features, which is also represented by the moderate invasiveness that is observed in Mlow-80028 patternoids (Fig. 2D–I).

***M-16992*** cells exhibit a fully mesenchymal phenotype characterized by the absence of E-cadherin, with expression levels of N-cadherin and vimentin similar to those observed in the intermediate *Mlow-8028* cells (Fig. 2A and C). This lack of E-cadherin eliminates any residual epithelial adhesion, allowing these cells to fully adopt a mesenchymal state (Fig. 2B), enhancing their invasive potential. The presence of N-cadherin and vimentin facilitates dynamic cell–ECM interactions and provides structural flexibility, supporting effective migration and invasion through the ECM resulting in the invasive phenotype of *M-16992* patternoids (Fig. 2D–I).

These findings reveal, that distinct invasive phenotypes correlate with the EMT-related molecular profiles of each PDAC subtype (Fig. 2B and D). The mesenchymal subtype *M-16992* demonstrates a large and complex invasive morphology, with a mean invasive area of 23 971 ± 102 507 μm^2^ (Fig. 2E), a maximum invasive distance of 379.7 ± 96.1 μm (Fig. 2G), and a branching factor of 3.15 ± 0.965, indicating an invasive complexity approximately two times greater than that of the epithelial (*E-9591*) and hybrid EMT (*Mlow-8028*) subtypes (Fig. 2H and I). The absence of E-cadherin in *M-16992* (Fig. 2A–C), which reduces cell–cell adhesion, likely facilitates the fractal development of its invasive branches (Fig. 2D, H and I).

In contrast, the cytoplasmic E-cadherin in *Mlow-8028* may support collective invasion, resulting in a lower branching factor of 1.61 ± 0.530 (Fig. 2D, H and I), comparable to the 1.56 ± 0.507, that is observed in the epithelial subtype. Despite its less complex structures (Fig. 2D), *E-9591* exhibits a notably high number of initial invasive events, averaging 7.5 ± 3.725 (Fig. 2H), which are evenly distributed along the microcavity boundaries (Fig. 1D). Meanwhile, *Mlow-8028* and *M-16992* display fewer initial invasive events, averaging 4.15 ± 2.492 and 4.8 ± 1.699 (Fig. 2H), yet these events lead to distinct morphologies: thicker branches in *Mlow-8028* and thinner, more fractal branches in *M-16992* (Fig. 2D). Moreover, all three PDAC subtypes exhibit distinct normalized non-invasive areas, which increase as the EMT score decreases (Fig. 2F). The inverted normalized non-invasive area can thus be interpreted as an indicator of invasiveness.

The relative EMT marker expression levels in patternoids of the PDAC subtypes *Mlow-8028* and *E-9591* do not differ significantly. Both subtypes show elevated levels of mesenchymal markers (N-cadherin and vimentin), albeit lower than in *M-16992*, and exhibit minimal E-cadherin expression (Fig. 8). These findings suggest that patternoids shift toward a mesenchymal phenotype either during invasion or as a prerequisite for matrix invasion, indicating the potential *in vivo* plasticity of PDAC.^15,16^

### 2.3 Development and invasion dynamics of PDAC patternoids

**Fig. 3:**
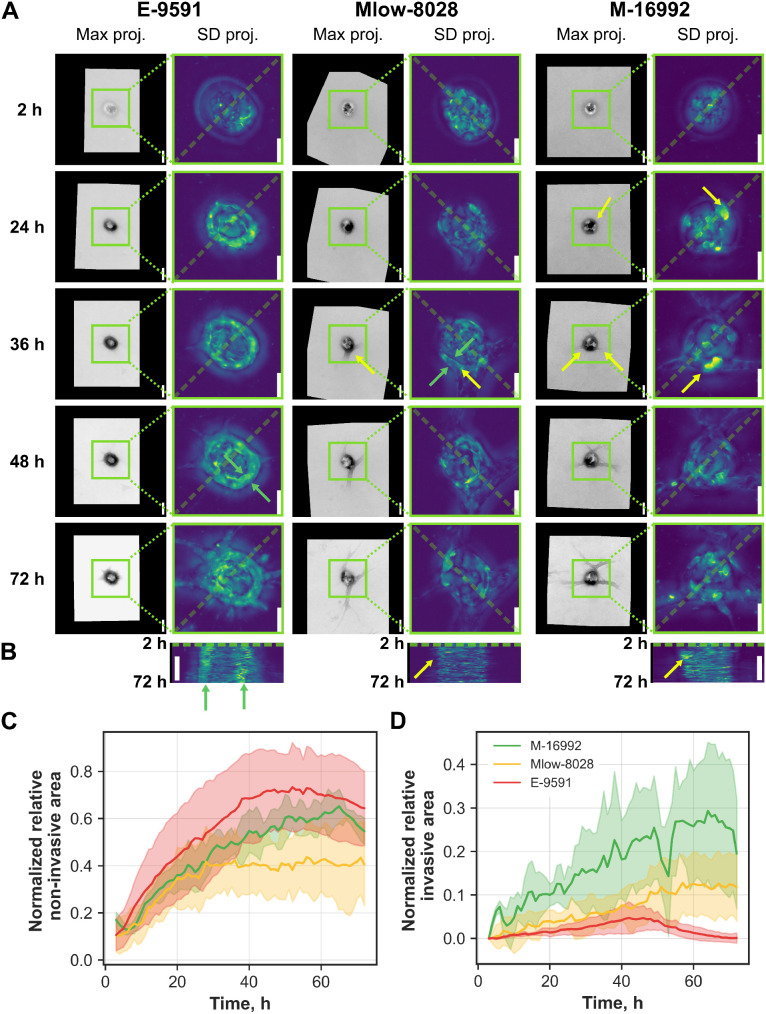
Invasion dynamics of PDAC subtype patternoids. A maximum projections of representative replicates of different PDAC subtypes patternoids at time points 2, 24, 36, 48, and 72 h (scale bar = 100 μm) with corresponding zoom-ins of the microcavity region (green boxes). For better visibility, standard deviation projections are shown (scale bar = 50 μm). Epithelial monolayer formation is observed in *E-9591*, while partial monolayer closure is seen in *Mlow-8028*. Invasion correlates with sites of cell accumulations in *Mlow-8028* and *M-16992* (yellow arrows). B Kymographs highlight invasion dynamics over 72 h, showing cohesive growth in *E-9591* (green arrows) and fluctuating migratory cells in *Mlow-8028* and *M-16992* (yellow arrows) (scale bar = 50 μm). C and D The relative quantification of the invasive and non-invasive area of PDAC patternoids derived from *c*_0_ class = 20 over time illustrates the subtype specific growth dynamics. Number of patternoid replicates used for analysis: *N* = 4 (E), *N* = 5 (Mlow) and *N* = 6 (M).

In order to investigate the temporal dynamics of patternoid invasion during the 3 day cultivation period leading up to the end-point analysis. To achieve this, patternoids from each PDAC subtype were imaged hourly over a 72 hour period. For consistency, quantification was limited to patternoid replicates initiated with a starting cell number *c*_0_ = 20. Representative replicates highlight the key developmental stages of patternoid formation for each PDAC subtype (see Fig. 3A). Careful examination of pre-invasion behavior within the microcavity not only provided insight into the “non-invasive area” parameter but also clarified the distinct invasive mechanisms of each PDAC subtype.

Upon seeding, cells transitioned from a rounded, non-adherent morphology to an adherent state along the microcavity walls, marking the onset phase. During this period, cells engaged with the collagen matrix and formed initial attachments, establishing structures that later drove subtype-specific invasion.

The quantitative and statistical analysis shows that the baseline values for the relative non-invasive area did not differ significantly across subtypes (Fig. 3C). This confirms that the initial condition, provided by a defined starting cell number of *c*_0_ = 20, were consistent across all patternoid replicates used in the analysis. Quantification of the non-invasive area revealed a statistically significant increase over time for all subtypes (Fig. 3C). Despite inherent limitations in brightfield quantification—such as potential inaccuracies in ROI alignment and illumination artifacts—the observed trends were reproducible across biological replicates. The slower expansion of the non-invasive area observed in both mesenchymal subtypes was significantly different from that of the epithelial subtype (*p* = 0.028 for *M-16992*; *p* < 0.001 for *Mlow-8028*; Fig. 3C). The small, non-significant difference in non-invasive growth rates between *M-16992* and *Mlow-8028* aligns with their qualitatively similar non-invasive morphologies shown in Fig. 3A.

The epithelial subtype *E-9591* displayed the steepest non-invasive growth trajectory, followed by *M-16992*, whereas *Mlow-8028* exhibited the slowest expansion, plateauing at approximately 40% after 27 h (Fig. 3C). Concurrently, the invasive area of *Mlow-8028* began to increase more steeply after 27 h (Fig. 3D), indicating a shift from proliferation within the microcavity to outward invasion (Fig. 3A). This subtype migrated as cohesive clusters, extending multicellular invasive strands (Fig. 3A), a behavior likely enabled by hybrid EMT traits such as partial cell–cell adhesion and moderate MMP expression (Fig. 2B).

A comparable shift occured in *E-9591* patternoids at around 38 hours, when the non-invasive area reached ∼70% (Fig. 3C). Following the formation of a continuous epithelial monolayer along the microcavity wall (Fig. 3A and B, green arrows), *E-9591* initiated cohesive, collective invasion through evenly distributed protrusions. The decline in the slope of the non-invasive area trajectory (Fig. 3C), together with a modest but detectable increase in invasive area (Fig. 3D), suggests that *E-9591* cells initiate invasion primarily in response to spatial confinement—consistent with their epithelial phenotype (Fig. 2B).

In contrast, *M-16992* showed an early and continuous increase in invasive area (see Fig. 3D), largely independent of its non-invasive growth trajectory. This behavior reflects a reduced reliance on spatial cues and is evident in the pronounced, branched morphology of its invasive front (see Fig. 3A) and its comparatively large relative invasive area (Fig. 3D and 2E). These features are characteristic of a strongly mesenchymal phenotype with high matrix remodeling capacity (Fig. 2B).

In summary, *E-9591* exhibits an initial phase of epithelial expansion followed by delayed, spatially regulated invasion; *Mlow-8028* demonstrates earlier invasion with hybrid characteristics; and *M-16992* demonstrates early, extensive, and spatially unconfined invasion typical of a mesenchymal program. These distinct invasion patterns reflect underlying EMT states and underscore the functional heterogeneity among PDAC subtypes.

### 2.4 Effect of starting cell number on invasion phenotypes

**Fig. 4:**
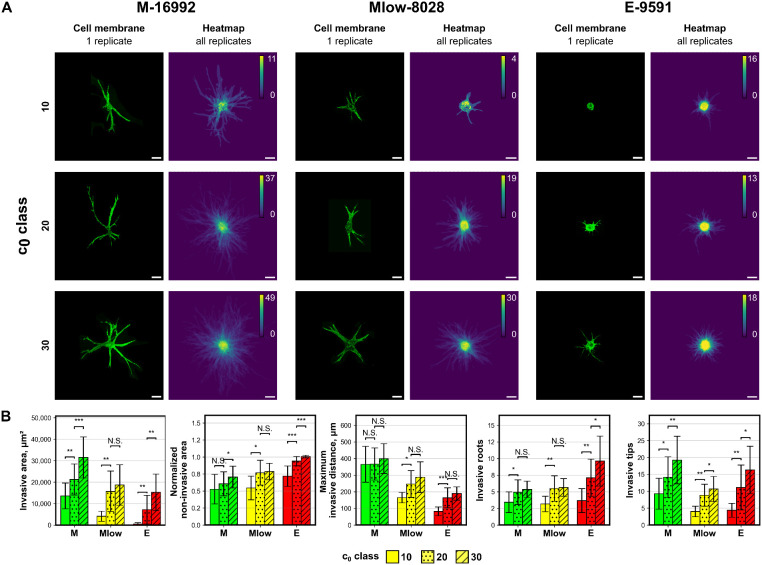
Distinct invasion phenotypes across different *c*_0_ classes for all PDAC subtypes. A Representative maximum projections *M-16992*, *Mlow-8028*, and *E-9591* patternoids (stained with CellMask-DeepRed membrane dye) and binary image heatmaps of all replicates that were used for the quantitative analysis in B. Initial cell numbers (*c*_0_) = 10, 20, and 30 were examined for their effect on invasive morphology. Scale bar: 100 μm. B Quantitative comparisons show distinct effect of *c*_0_ classes on invasive parameters. *N* = 2 independent experiments.

Building on the observation that the initial confluency is a critical factor for invasion onset, its influence was systematically assessed across the different PDAC subtypes. Invasive parameters were quantified for patternoids derived from *c*_0_ classes of 10, 20, and 30 to evaluate the role of cell density in shaping invasion phenotypes (Fig. 4A and B).

Across all subtypes, higher *c*_0_ classes resulted in a consistent and significant increase in invasive and non-invasive area, maximum invasive distance, and branching factor (Fig. 4B). Despite intrinsic differences in invasive propensity among the subtypes, these trends remained conserved, underscoring the importance of confluence for invasion, irrespective of the PDAC subtype and the corresponding invasive mechanisms (Fig. 9).

**Fig. 5:**
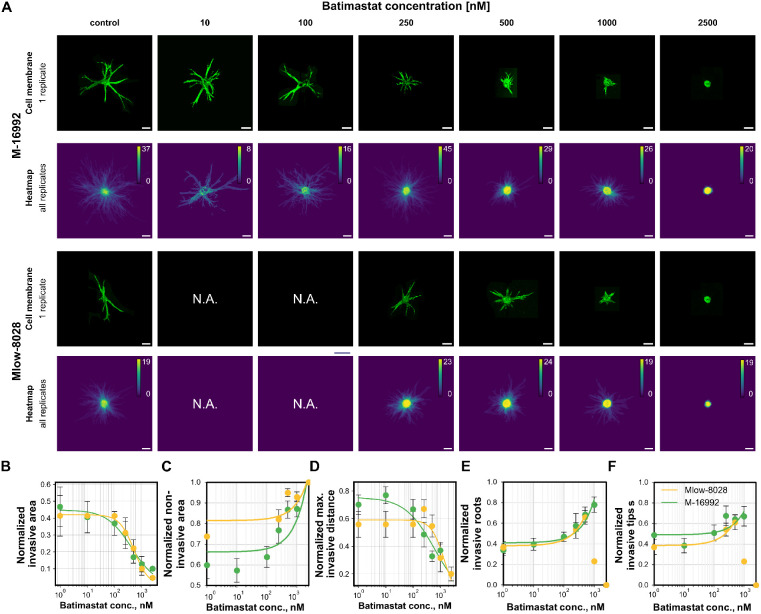
Batimastat dose–response effects on PDAC patternoid invasiveness. A Representative maximum projections of patternoids for *M-16992* and *Mlow-8028* (stained with CellMask-DeepRed membrane dye) and binary image heatmaps of all replicates that were used for the quantitative invasion analyses. B The invasive area (μm^2^) decreases with increasing batimastat concentrations, following a Hill-fit. *M-16992* exhibits a steeper decline and an 18% lower EC_50_ = 424.50 compared to *Mlow-8028* (EC_50_ = 521.15). C Normalized non-invasive area increases, plateauing at 500–1000 nM for both subtypes. *Mlow-8028* exhibits a flatter slope (*m* = 0.00005) compared to *M-16992* (*m* = 0.002), reflecting a slower inhibitory response to batimastat. D Maximum invasive distance (μm) decreases by >80% at 1000 nM for both subtypes, indicating protease inhibition-driven shifts. *M-16992* shows a steeper decline with an EC_50_ of 629.74 nM. *Mlow-8028* exhibits a 2.26 times higher EC_50_ of 1424.11 nM, reflecting reduced sensitivity to batimastat treatment. E and F Normalized invasive roots and tips as a linear function of batimastat concentration. Both subtypes show a gradual increase in invasive roots and tips, with slightly reduced branching complexity, indicative of spatial-constraint-induced invasion with epithelial-like characteristics. A linear fit was applied to the data below 500 nM, as data above this concentration was excluded due to offset effects. No significant difference is observed between subtypes.

The findings establish the starting cell number *c*_0_ as a critical experimental parameter for controlling invasion phenotypes and ensuring reproducibility. Accurate documentation of *c*_0_ across experiments is essential for reliable phenotypic characterization and robust assessment of therapeutic interventions across multiple batches (Fig. 5).

### 2.5 Drug sensitivity assay for invasion mechanisms

We next sought to demonstrate the potential of the PDAC patternoid model for detailed phenotypic drug testing, aimed at further investigating protease- and spatial constraint-mediated invasion mechanisms in the mesenchymal subtypes *Mlow-8028* and *M-16992* (Fig. 5). By leveraging the reproducible patternoid formation achieved through collagen-based microcavity arrays, we performed a drug sensitivity assay to distinguish between protease-mediated and spatial constraint-driven invasion mechanisms in these subtypes. The assay workflow—including cell seeding, patternoid formation, drug treatment, and endpoint analysis—was optimized for consistency, as described earlier.

Batimastat, a broad-spectrum matrix metalloproteinase (MMP) inhibitor, was used to suppress protease-mediated invasion, enabling us to observe the phenotypic shifts in response to drug treatment across increasing concentrations (10–2500 nM). Dose–response analyses revealed notable trends across the two subtypes, highlighting their distinct reliance on protease-dependent invasion. Quantitative invasion parameters—including invasive and non-invasive area, maximum invasive distance, number of invasive roots and tips—were extracted and analyzed using a Hill model where applicable, to capture dose-dependent responses. Otherwise linear fits were applied to identify residual linear trends.

As shown in Fig. 5, batimastat induced a concentration-dependent reduction of invasive features in both M-16992 and Mlow-8028, albeit with distinct sensitivities. The invasive area was strongly reduced in both subtypes, following a sigmoidal dose–response curve (Fig. 5B). *M-16992* exhibited a lower EC_50_ (424.5 nM) compared to *Mlow-8028* (521.2 nM), indicating higher sensitivity to batimastat. The normalized non-invasive area increased proportionally with batimastat concentration, plateauing between 500 and 1000 nM, indicating a progressively reduced capacity of cells to escape the microcavity. This reflects a concentration-dependent stabilization of the confined patternoid body under MMP inhibition. Notably, the steeper slope observed for *M-16992* (*m* = 0.002) compared to *Mlow-8028* (*m* = 0.00005) highlights a more abrupt cessation of invasion upon protease inhibition, consistent with its higher batimastat sensitivity reflected in the invasive area reduction (Fig. 5C). The maximum invasive distance decreased by over 50% already at 500 nM batimastat, reflecting an effective impairment of long-distance invasion (Fig. 5D). Again, *M-16992* showed a markedly lower EC_50_ (629.74 nM) than *Mlow-8028* (1424.11 nM), confirming a stronger protease dependency for *M-16992* in regards of invasive front progression. Both subtypes exhibited up to a twofold increase in invasive roots and a moderate rise in invasive tips at intermediate batimastat concentrations (500 nM), followed by saturation or reduction at higher doses (≥1000 nM) (Fig. 5E and F). These trends reflect a shift toward less branched invasion modes, with an increased number of initial invasion events. This behavior mirrors the invasion adaptation previously observed for epithelial subtypes under spatial confinement (Fig. 2D and E), before offset effects were observed for concentrations ≥1000 nM. No significant differences between the two subtypes were observed for these parameters.

Both subtypes showed a consistent adaptive response to MMP inhibition characterized by an increase in invasive roots and reduced branching complexity (Fig. 5E and F)—morphologically resembling the invasive phenotype of epithelial subtypes (Fig. 3A–C). Since batimastat does not affect proliferation, the observed adaptation is likely a consequence of increased spatial constraints and mechanical compression within the microcavity, as previously described for the epithelial subtype (Fig. 3A and C). Under these conditions, cells initially attempt to maintain invasive escape mechanism by increasing the number of protrusions (invasive roots), but ultimately fail to penetrate the matrix once proteolytic activity is fully blocked. The resulting epithelial-like invasion, characterized by significantly reduced invasive distance and area, reflects a transition to spatial confinement-driven invasion mechanisms. This suggests a pronounced plasticity of PDAC subtypes and their ability to dynamically adapt to protease inhibition and environmental constraints.

## 3 Conclusion

This study presents a 3D patternoid platform that enables detailed phenotypic analyses of PDAC invasion mechanisms while addressing key limitations of existing 3D culture systems. Conventional PDO systems, while successful in generating chemograms and establishing drug-response correlations, face challenges in reproducibility due to uncontrolled starting cell numbers, reliance on viability assays, and oversimplified environments that fail to capture invasive phenotypes. By employing biomimetic microcavities, the patternoid platform standardizes organoid formation, allowing for controlled starting conditions and reproducible phenotypic analyses.

Our findings demonstrate that the patternoid system exhibits sensitivity in correlating epithelial-to-mesenchymal transition (EMT) scores with distinct invasive phenotypes, despite the shift of epithelial and hybrid PDAC subtypes toward mesenchymal-like subtypes during patternoid development. This observation affirms the invasive potential of different PDAC subtypes while accounting for PDAC plasticity in a tumor-like microenvironment.

Moreover, integrating EMT scores with invasion dynamics through a multi-parameter classification approach can improve the stratification of PDAC subtypes. This co-classification allows for a more refined analysis by not only considering the molecular EMT status but also incorporating functional aspects such as migration patterns, protease dependency, and spatial constraints, which themselves vary with the initial cell number. Such an approach enhances the predictive power of the system, ensuring a more comprehensive understanding of tumor cell behavior and its dependence on experimental conditions.

Live-cell measurements, as depicted in Fig. 3, provide additional insights into temporal invasion dynamics, paving the way for real-time analyses, particularly for applications involving other cell populations like patient-derived organoids (PDOs). Further refinement of the system to better mimic the *in vivo* tumor microenvironment (TME)—for instance, by incorporating co-culture with immune cells to study tumor-immune interactions or dynamic microenvironments that simulate fluid flow, mechanical stress, or extracellular matrix remodeling—could significantly expand its utility. Additionally, the platform's versatility could be increased by extending its applications to other solid tumors and incorporating advanced readouts, such as real-time metabolic profiling or immune cell interactions.

Drug sensitivity assays further demonstrated the platform's ability to distinguish between protease-mediated and spatial constraint-driven invasion mechanisms. Batimastat treatment confirmed that MMP activity predominantly drives invasion in mesenchymal subtype *M-16992* in comparison to the hybrid EMT subtype *Mlow-8028*. Dose–response analyses revealed complementary invasion strategies, with *M-16992* displaying higher sensitivity to protease inhibition, transitioning to an epithelial-like phenotype at lower drug concentrations compared to *Mlow-8028*.

Currently, the primary bottleneck limiting the platform's scalability, is the reliance of classical tools, such as CellPose, on high-quality images—particularly in bright-field microscopy—to identify cell segments and, consequently, determine cell numbers. This reliance results in a trade-off between the scan time of the gel overview and the image resolution, which restricts the number of samples that can be scanned per experiment before cells begin forming clusters or proliferating. However, this limitation could be addressed in the future through the application of machine or deep learning-based image processing tools, which do not depend on high-resolution images for cell number determination. In the context of deep learning, the ability to generate a high number of replicates through parallelization offers substantial potential for creating robust training and testing datasets. This capability is particularly valuable for applications such as the high-throughput analysis of drug responses.

In summary, the 3D patternoid platform offers a reproducible and adaptable system for studying cancer invasion mechanisms. Its innovative design, which standardizes organoid generation and addresses inter-organoid reproducibility challenges, represents a major advancement in cancer modeling. Future efforts to optimize its components and broaden its applications may further solidify its role in personalized medicine and preclinical studies.

## 4 Methods

### Collagen microcavity preparation

Biomimetic microcavities were fabricated to cultivate pancreatic ductal adenocarcinoma (PDAC) patternoids using a polydimethylsiloxane (PDMS) stamp-assisted molding technique, as previously described.^17^ For this, a solution of rat tail collagen type I (ibidi) was prepared following the manufacturer's protocol. To mimick the elevated stiffness characteristic of the tumor microenvironment, a collagen concentration of 5 mg mL^−1^ was selected, although the method is compatible with concentrations ranging from 2 to 6 mg mL^−1^.

The collagen solution was adjusted to physiological pH and degassed to eliminate air bubbles. A custom-designed PDMS stamp was then used to create a precise array of 25 × 26 cylindrical microcavities in the collagen matrix. The microcavities were defined by a diameter of 100 μm, a height of 200 μm, and a spacing of 600 μm between centers. The collagen solution was polymerized under the PDMS stamp at 37 °C for 90 min, ensuring stable cavity formation.

After polymerization, the PDMS stamp was carefully removed, resulting in a patterned collagen gel. The patterned gels were then immersed in cultivation medium to maintain hydration during storage at 4 °C under sterile conditions. Prior to cell seeding, the cultivation medium was removed.

### Cell sources and classification

Primary cancer cells were isolated from a genetically engineered Kras (G12D) mouse model. The cells *M-16992*, *Mlow-8028* and are three distinct tumor-derived populations, that were characterized *via* RNA-Seq and were classified based on Kras mutation dosage and EMT scores.^12^ The correlation between Kras dosage and EMT score reflects key aspects of molecular heterogeneity observed in human PDAC subtypes. Consequently, it is reasonable to refer to these cells as PDAC subtype-derived in the context of this study. The cells were generously provided by Saur and Reichert.

### Cell and patternoid maintanance

Cells were initially thawed from cryopreserved stocks by incubating at 37 °C until a small ice particle remained in the suspension. The thawed suspension was transferred into 10 mL of prewarmed Dulbecco's modified Eagle medium (DMEM) supplemented with 10% fetal bovine serum (FBS) and 1% penicillin–streptomycin (P/S). Cells were centrifuged at 300 × *g* for 5 min, the supernatant was discarded, and the cell pellet was resuspended in fresh medium. Cells were cultured as adherent monolayers in T175 culture flasks to generate sufficient cell numbers for the experiments under standard physiological conditions (37 °C, 5% CO_2_ in a humidified atmosphere). Confluency was monitored regularly, and cells were split when reaching 70–90% confluency. Splitting involved trypsinization using trypsin–EDTA for 5 min at 37 °C, followed by neutralization with fresh medium, centrifugation to pellet the cells, resuspension in fresh medium, and cell counting to determine density for seeding. Routine checks for contamination, including mycoplasma testing, and maintaining a sterile environment were performed to ensure reproducibility. For experimental consistency, all cells were harvested at the same confluency and maintained under the same number of splitting cycles post-thaw, although passage numbers varied between the different PDAC subtypes (*M-16992* P21, *Mlow-8028* P25, *E-9591* P28).

For cell monolayer experiments, the cells were seeded into ibidi 4-well imaging dishes at single cell densities and cultivated under standard culture conditions for 24 h before further processing for imaging.

For the generation of patternoids, the harvested cells were seeded into the prepared microcavities at a cell density of 0.5 × 10^7^ cells per mL and allowed to settle for 2 min at room temperature to ensure even distribution. Excess cells were washed away, and a thin layer of collagen was added to create a closed 3D extracellular matrix (ECM) environment. For that, a coverslip was covered with a thin layer of collagen solution and placed on top of the collagen gel. The cover slip was removed carefully after collagen polymerization. Fresh medium was added and the cells were incubated under standard culture conditions for 72 h. A medium change was performed once after 24 h for both control and drug treatment experiments. During this period, cells adhered, proliferated, and formed subtype-specific invasive structures suitable for further analysis.

### Fluorescent staining and imaging

After 72 h of cultivation, PDAC patternoids were fixed and stained for fluorescence imaging. To ensure adequate staining within the dense collagen matrix, the collagen gel was pre-digested with collagenase for 5 min at 37 °C and 70 rpm. During this step, membrane staining for endpoint analysis was performed with a 1 : 1 dilution of CellMask DeepRed (1× in DMEM) and collagenase. The digestion reaction was terminated by washing with ice-cold phosphate-buffered saline (PBS), followed by fixation with 4% paraformaldehyde (PFA) for 30 min at room temperature (RT) and 70 rpm. After thourough washing with PBS, the nucleus staining with 10 μg mL^−1^ Hoechst 33342 was performed for 30 min at RT and 60 rpm. Subsequently, the samples were washed with PBS and stored at 4 °C until imaging within 24 h for nucleus segmentation and within 7 days for patternoid segmentation.

Immunofluorescent staining was conducted on PDAC subtype cell monolayers and patternoids to analyze epithelial-to-mesenchymal transition (EMT) markers, including E-cadherin, N-cadherin, and vimentin, for subtype characterization. Cell membranes were permeabilized using 0.1% Triton-X for 30 min at RT, followed by blocking with serum from the secondary antibody donor species overnight at 4 °C. Both primary and secondary antibodies were prepared in PBS supplemented with 5% bovine serum albumin (BSA). Primary antibodies were incubated overnight at 4 °C, and after thorough PBS washes, secondary antibodies were incubated for 2.5 h at RT. Stained samples were stored in PBS at 4 °C and imaged within 24 h.

### EMT score analysis in cell monolayers and patternoids

Immunofluorescently stained samples were imaged using a confocal fluorescence microscope. Secondary antibody controls, prepared without primary antibodies, were utilized to optimize imaging parameters and minimize background or nonspecific signal detection. Uniform imaging settings were applied across all three subtypes to ensure comparability.

Confocal images were processed to generate a maximum z-projection. For single-cell and patternoid segmentation, the E-cadherin channel was used for the epithelial subtype *E-9591*, while the N-cadherin channel was employed for the mesenchymal subtypes *M-16992* and *Mlow-8028*. The mean fluorescence intensity of the vimentin, E- and N-cadherin channels was measured within the segmented areas to quantify cytoplasmic expression levels per cell/patternoid.

### Patternoid selection and imaging workflow

**Fig. 6:**
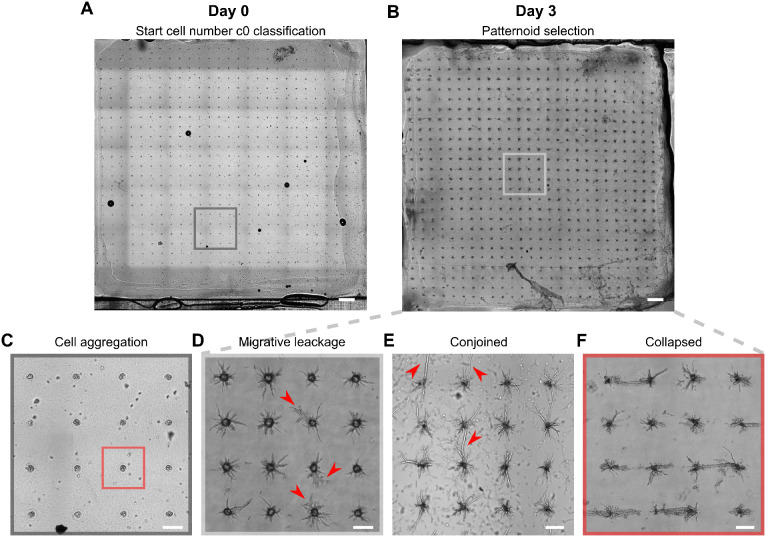
Representative overview scans of patterned collagen gels at quality control steps on day 0 and day 3, along with examples of patternoids that were excluded from analysis. A Representative overview scan of the complete collagen gel at day 0, required for c0 classification and quality control of microcavity quality for each patternoid ID. B Representative overview scan of the complete collagen gel at day 3, required for quality control of patternoid development (here: E-9591). C and D Corresponding zoom-in views of (A) and (B). The red box in (C) highlights a microcavity containing a cell agglomeration, which prevents proper single-cell counting for determining the initial cell number; thus, it is excluded from analysis. In (D), red arrows indicate planar outgrowth of cells, which suggests migratory rather than invasive behavior due to collagen lid detachment from the collagen gel—hence excluded from analysis. E Red arrows highlight the conjoinment of neighboring M-16992 patternoids at day 3, leading to their exclusion from analysis. F Collapsed 3 days old M-16992 patternoid structures due to collagen lid degradation, caused by improper collagen digestion required for cell membrane staining (*e.g.*, insufficient stopping of digestion on ice), resulting in their exclusion from analysis.

An overview image of each collagen gel sample was captured within 2 h after cell seeding (Fig. 6A), before single cells began to merge (Fig. 6C), to determine the initial cell number in each microcavity. Imaging was performed using an epifluorescence microscope, and initial cell numbers were assigned to individual patternoids based on their positional IDs within the matrix. Patternoids derived from *c*_0_ classes of 10, 20, and 30 cells (±10%) were used for analysis.

**Fig. 7:**
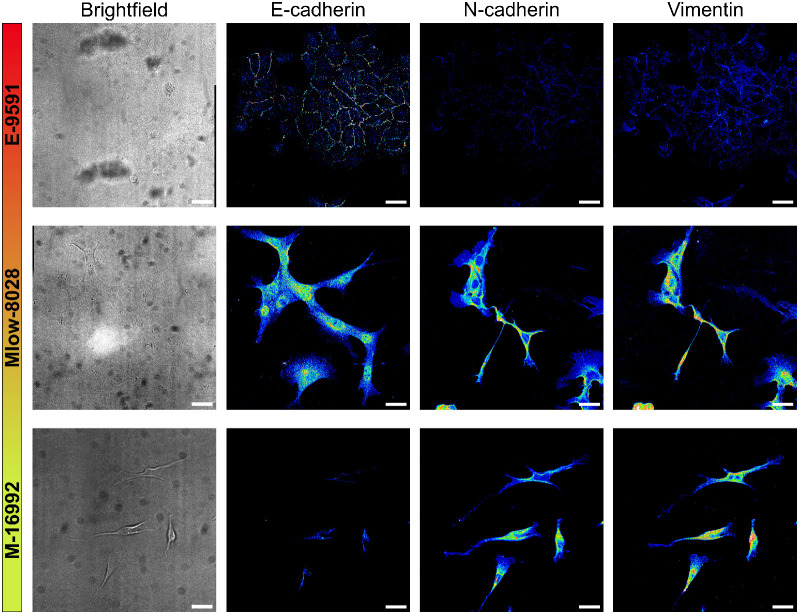
Visulization of epithelial and mesenchymal marker expression across PDAC subtypes. Immunofluorescence and corresponding brightfield images of E-9591, Mlow-8028 and M-16992, stained for E-cadherin, N-cadherin, and vimentin. To improve visibility and ensure consistent comparison across subtypes, the intensities for N-cadherin and vimentin were not adjusted in the fluorescence images in Fig. 2, as this would have caused oversaturation in Mlow-8028 and M-16992 due to their high expression levels. To allow visualization of low expression in E-9591, an alternative representation using a 16-color lookup table (LUT) with a reduced dynamic range (16 instead of 255) were applied. Scale bar = 50 μm.

**Fig. 8:**
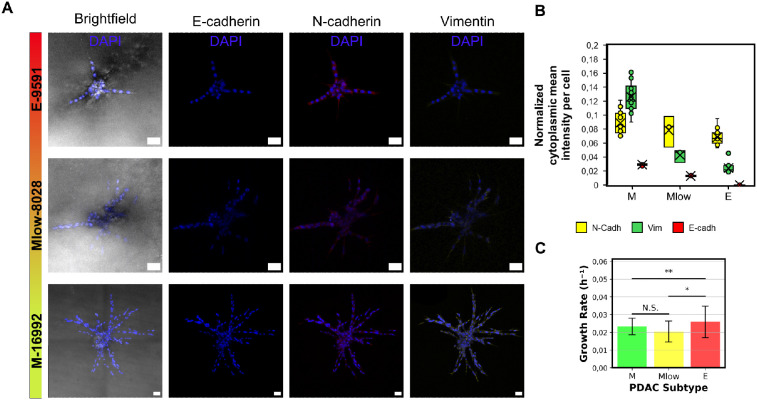
Expression of EMT markers and growth rates in PDAC subtype patternoids. A Brightfield images of representative patternoid replicates and maximum projections of fluorescence channels for EMT markers, along with DAPI (blue) for nuclear localization. Scale bar = 50 μm. B Cytoplasmic expression levels of EMT markers in different PDAC subtype patternoids after three days of cultivation. C Growth rates of different PDAC subtypes (mean of all patternoid replicates used in batch 2 of patternoid characterization analyses, with standard deviation indicated by error bars). Significant differences were observed between mesenchymal (M and Mlow) and epithelial PDAC subtypes.

**Fig. 9:**
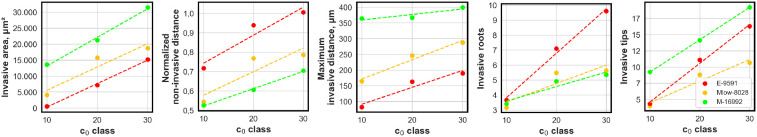
Effect of *c*_0_ class on invasive phenotypes across PDAC subtypes. Trends for invasive area (μm^2^), normalized non-invasive area, maximal invasion distance (μm), branch tips, and initial branches are shown for the PDAC subtypes *E-9591*, *Mlow-8028*, and *M-16992*. Each data point represents the mean value for a given *c*_0_ class (10, 20, and 30), with dashed lines indicating the trendlines for each subtype. Statistical analysis revealed no significant differences (N.S.) in the degree of dependence on *c*_0_ class between the PDAC subtypes *M-16992 vs. Mlow-8028* and *Mlow-8028 vs. E-9591* for any of the evaluated invasive parameters, except for “invasive roots” for *E-9591 vs. Mlow-8028*/*M-16992*. This highlights the importance of documenting and maintaining comparable distributions of *c*_0_ classes across experimental conditions to ensure reliable interpretation of subtype-specific invasion phenotypes.

For drug treatment experiments, an additional overview scan of each sample was conducted immediately before the addition of batimastat.

The final overview scan was performed post-fixation and staining to serve as a quality control measure and as a basis for selecting patternoids suitable for further analysis (Fig. 6B). Patternoid selection criteria included the following:

**Day 0:**

• Successful identification of the starting cell number with no evidence of cell merging in the initial overview scan (Fig. 6C).

• Absence of excess cells surrounding the patternoids that could interfere with final patternoid formation (Fig. 6C).

**Day 1 (drug treatment experiments only):**

• Patternoids classified as non-invasive at the time of drug addiction.

**Day 3:**

• Successful cell membrane staining for segmentation during analysis.

• No conjoined neighboring patternoids throughout the cultivation period (Fig. 6E).

• Presence of invasive cells only, without planar cell growth between the collagen gel and lid (indicative of migratory leakage or improperly sealed regions, Fig. 6D) and no collapsed structures caused by collagen pre-digestion (Fig. 6F).

A selection of as many patternoid replicates as possible was made based on these criteria, aiming for approximately 20 replicates per *c*_0_ class. For the *c*_0_ = 10 class, this target was not achievable due to boundary condition constraints.

Selected replicates were imaged using a Leica confocal microscope Stellaris 8 equipped with a WLL. Imaging was performed with a Zeiss objective (32× magnification, 0.4 NA, water immersion) and an LHC PL FLUOTAR objective (10× magnification, 0.3 NA, dry). Imaging parameters included z-step sizes of 2 μm and 5 μm, pixel sizes of 0.569 μm and 2.27 μm, and gain values of 100 and 25, respectively. Constant parameters across both setups ensured comparability, including a frame rate of 1 frame per s, laser power at 1%, scan speed of 400 Hz, image resolution of 512 × 512 pixels, and use of the HyD X3 detector.

Images acquired under these conditions were processed using a custom analysis pipeline implemented as a macro in *ImageJ Fiji*. The pipeline included the following steps:

1. **Maximum z-projection:** consolidated three-dimensional imaging data into two-dimensional formats.

2. **Manual cropping:** patternoids extending beyond the field of view were cropped to standardize the dataset.

3. **Denoising:** background noise was reduced to enhance structural clarity.

4. **Binary image generation:** processed data were converted into binary images for further analysis.

Standardized sample preparation protocols enabled the application of consistent thresholds for binary image generation. Minor manual adjustments to thresholds were made as needed to address sample variability. The binary images were then analyzed to extract the following parameters:

• **Invasive area:** total area infiltrated by cells beyond the microcavity boundary.

• **Normalized non-invasive area:** fraction of the cell-covered microcavity area normalized to the total microcavity area with *A* = 7854 μm^2^.

• **Maximum invasive distance:** the farthest distance cells invaded into the collagen matrix.

• **Branching ratio:** ratio of the invasive roots and tips.

Dose–response experiments with batimastat were analyzed by fitting the data to the Hill equation, enabling the calculation of EC_50_ values.

### Growth rate measurement

The same patternoids that were used for invasive parameter analyses, identified by a unique patternoid ID and its initial starting cell number *c*_0_, underwent subsequent quantification analysis of the final cell number after 72 h of cultivation. For the nucleus segmentation, a high-resolution fluorescent nucleus signal with minimal background noise and small z-stack step size was required. The segmentation was performed in Arivis software. The quantified cell numbers were used to compute the growth rate (*r*) using the exponential growth model:

1 *c*(*t*) = *c*_0_·*e*^*rt*^,

where *c*(*t*) is the cell count at time *t* (here: 72 h), *c*_0_ is the initial cell count, and *r* represents the exponential growth rate.

### Statistical analysis

To evaluate statistically significant differences between PDAC subtypes, *c*_0_ classes, or replicates from different experimental batches, two-sided Student's *t*-tests were performed using Python (version 3.10.2) with the scipy.stats module. For all comparisons, an unpaired two-tailed Student's *t*-test was conducted, assuming equal variances between groups.

Results are presented as mean ± standard deviation (SD). Sample sizes (*n*) represent independent biological replicates, with technical replicates averaged prior to statistical analysis to avoid pseudoreplication. A significance level of *α* = 0.05 was used, and *p*-values below this threshold were considered statistically significant.

### Quantification and statistics of non-invasive and invasive area over time

Due to the limitations of brightfield imaging, such as insufficient contrast for precise segmentation and the lack of fluorescence markers in the dynamic phase, conventional binary masks could not be generated for time-resolved patternoid analysis. To enable quantification of invasion dynamics, we established a standardized method based on gray-value intensity measurements within predefined ROIs using Fiji/ImageJ.

Non-invasive and invasive areas were defined by concentric circular ROIs: the non-invasive area was measured within a 100 μm diameter circle centered on the patternoid, while the invasive area was calculated as the XOR between this ROI and a larger ROI encompassing the entire structure. Brightfield intensities within each ROI were normalized to a background measurement taken from a collagen-only region to correct for acquisition variability. This approach enabled robust measurement of relative changes in cell-covered area over time, which served as a proxy for dynamic tissue expansion. The invasive area was corrected relative to the signal at 3 h, such that all replicates started from a common baseline, enabling assessment of dynamic trends independently of minor imaging variability. ROIs were carefully aligned across timepoints using the StackRack plugin when possible; otherwise, fixed ROIs were applied and visually verified. Background ROI placement was consistent for all timepoints within a given replicate. To test for subtype-specific dynamics in invasive and non-invasive area development, we applied linear mixed-effects models (LME) using the statsmodels package in Python. Models were fitted separately for non-invasive (*Final* (*NInvA*)) and invasive (*Final* (*InvA*)) areas, with *Time*, *Subtype*, and their interaction as fixed effects, and *Replicate ID* as a random intercept to account for repeated measurements. This approach allowed us to assess differences in both baseline values and trends over time across subtypes.

## Author contributions

S. C. K., M. R. and A. R. B. designed research; S. C. K., V. C. H., T. F. T. and A. R. B. performed research; S. C. K. analyzed data; and S. C. K., M. R. and A. R. B. wrote the paper.

## Conflicts of interest

There are no conflicts to declare.

## Data Availability

The data supporting this article have been included as part of the ESI.†
